# UBE2J1 is the E2 ubiquitin-conjugating enzyme regulating androgen receptor degradation and antiandrogen resistance

**DOI:** 10.1038/s41388-023-02890-5

**Published:** 2023-11-29

**Authors:** Carla Rodriguez Tirado, Choushi Wang, Xiaoling Li, Su Deng, Julisa Gonzalez, Nickolas A. Johnson, Yaru Xu, Lauren A. Metang, Medha Sundar Rajan, Yuqiu Yang, Yi Yin, Mia Hofstad, Ganesh V. Raj, Song Zhang, Andrew Lemoff, Wei He, Jie Fan, Yunguan Wang, Tao Wang, Ping Mu

**Affiliations:** 1 Department of Molecular Biology, UT Southwestern Medical Center, Dallas, TX USA; 2 Quantitative Biomedical Research Center, Peter O’Donnell Jr. School of Public Health, UT Southwestern Medical Center, Dallas, TX USA; 3 Department of Urology, UT Southwestern Medical Center, Dallas, TX USA; 4 Harold C. Simmons Comprehensive Cancer Center, UT Southwestern Medical Center, Dallas, TX USA; 5 Peter O’Donnell Jr. School of Public Health, UT Southwestern Medical Center, Dallas, TX USA; 6 Department of Biochemistry, UT Southwestern Medical Center, Dallas, TX USA; 7 Accutar Biotechnology, Inc., Wilmington, DE USA; 8 Division of Biomedical Informatics, Cincinnati Children’s Hospital Medical Center, Cincinnati, OH USA; 9 Department of Pediatrics, University of Cincinnati, Cincinnati, OH USA; 10 Hamon Center for Regenerative Science and Medicine, UT Southwestern Medical Center, Dallas, TX USA

**Keywords:** Prostate cancer, Cancer therapeutic resistance, Molecular biology, Cancer genetics

## Abstract

Prostate cancer (PCa) is primarily driven by aberrant Androgen Receptor (AR) signaling. Although there has been substantial advancement in antiandrogen therapies, resistance to these treatments remains a significant obstacle, often marked by continuous or enhanced AR signaling in resistant tumors. While the dysregulation of the ubiquitination-based protein degradation process is instrumental in the accumulation of oncogenic proteins, including AR, the molecular mechanism of ubiquitination-driven AR degradation remains largely undefined. We identified UBE2J1 as the critical E2 ubiquitin-conjugating enzyme responsible for guiding AR ubiquitination and eventual degradation. The absence of UBE2J1, found in 5–15% of PCa patients, results in disrupted AR ubiquitination and degradation. This disruption leads to an accumulation of AR proteins, promoting resistance to antiandrogen treatments. By employing a ubiquitination-based AR degrader to adeptly restore AR ubiquitination, we reestablished AR degradation and inhibited the proliferation of antiandrogen-resistant PCa tumors. These findings underscore the fundamental role of UBE2J1 in AR degradation and illuminate an uncharted mechanism through which PCa maintains heightened AR protein levels, fostering resistance to antiandrogen therapies.

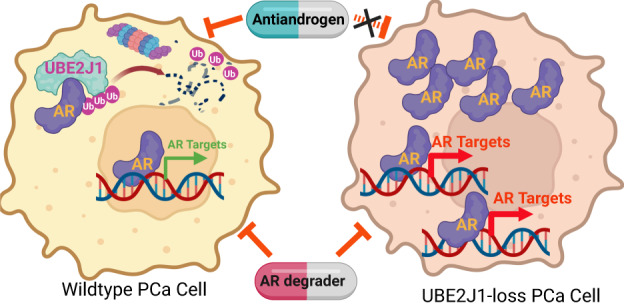

## Introduction

The androgen receptor (AR) is a crucial protein in human physiology, which significantly impacts various male and female physiological processes [1, 2]. As a nuclear transcription factor, AR interacts with testosterone, translocates to the nucleus, and regulates numerous genes, thereby influencing essential cellular events such as cell proliferation, differentiation, and apoptosis [3]. The importance of AR in human physiology becomes particularly evident in prostate cancer (PCa) where aberrant AR signaling emerges as a critical driver of tumorigenesis [4, 5]. Nevertheless, despite the enormous success with existing therapeutic strategies targeting AR and AR signaling pathways like antiandrogens, drug resistance presents a significant challenge, often limiting clinical outcomes and underscoring the urgent need for novel therapies to overcome this resistance [6–9]. A distinguishing characteristic of antiandrogen resistance is the persistence or enhancement of AR signaling observed in many resistant tumors [6, 10, 11], although the underlying molecular mechanisms remain largely unclear.

Ubiquitination-based protein degradation, a pivotal process in cellular functionality, prevents the accumulation of redundant and harmful proteins [12, 13]. Any dysregulation of this process can trigger an accumulation of oncogenic proteins, contributing to tumorigenesis and therapy resistance across various cancers [14, 15]. The intricate process of ubiquitination-dependent protein degradation involves careful coordination between E1 (activating), E2 (conjugating) and E3 (ligating) enzymes [16, 17]. Unlike other critical factors in protein degradation, E2 ubiquitin-conjugating enzymes determine the attachment location of ubiquitin on the target protein, thus dictating the fate of the protein [18, 19]. Importantly, E2 enzymes are vital for the ubiquitin-substrate specificity in the ubiquitination process [20]. Recent evidence suggests that dysregulation of E2 enzymes plays important roles in many aspects of tumorigenesis, including DNA repair, apoptosis, and oncogenic signaling pathways [20, 21]. Despite the integral role of E2 enzymes in tumorigenesis, the lack of insight into the specific E2 enzyme driving AR degradation poses a critical, yet unresolved, gap in the understanding of PCa and antiandrogen therapy resistance.

In this study, we identified UBE2J1 as the bona fide E2 ubiquitin-conjugating enzyme for AR ubiquitination in PCa. Our findings illustrate that the frequent loss of UBE2J1 in PCa, occurring in 5–15% of patients (cbioportal.org) [22, 23], dysregulates and impairs AR ubiquitination and degradation. This in turn leads to the accumulation of AR proteins, enabling UBE2J1-loss PCa cells to develop resistance to antiandrogen treatment. To counter this resistance, we employed a ubiquitination-based AR degrader to restore AR ubiquitination effectively, thereby inhibiting the growth of UBE2J1-loss PCa cells. These insights not only uncover the crucial role of UBE2J1 as the key E2 ubiquitin-conjugating enzyme for AR degradation but also illuminate an uncharted mechanism through which advanced PCa cells elevate AR protein levels to resist existing antiandrogen therapy. These insights could guide the development of more effective therapeutic strategies against advanced PCa.

## Results

### UBE2J1-loss confers resistance to antiandrogens

**Fig. 1:**
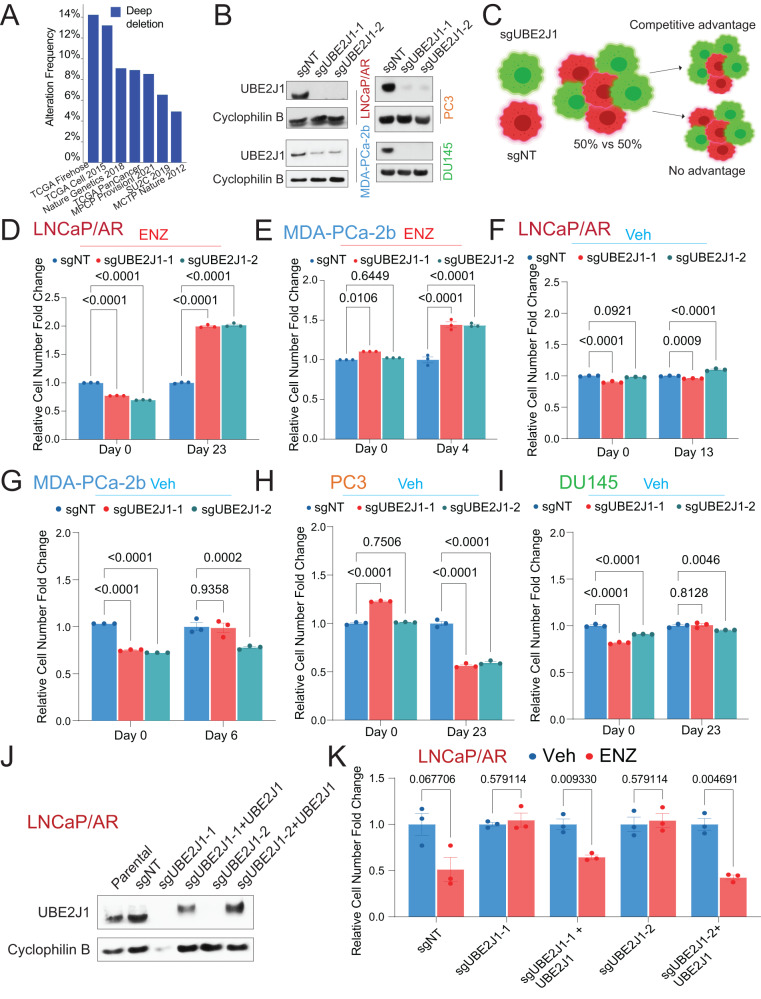
UBE2J1-loss promotes antiandrogen resistance in vitro. **A** Bar plot represents the frequency of UBE2J1-loss across multiple prostate cancer patient datasets, figure generated using cbioportal.org. **B** Western blots of UBE2J1 and Cyclophilin B proteins, demonstrating UBE2J1 KO in LNCaP/AR, MDA-PCa-2b, DU145 and PC3 prostate cancer cells transduced with Cas9 and annotated guide RNAs. **C** Representative schematic for fluorescence-activated cell sorting (FACS)-based competition assay. Schematic figure was created with BioRender.com. **D**–**I** Bar Plots represent the relative cell number fold change for FACS-based competition assay in (**D**) LNCaP/AR cells under 10 µM ENZ treatment, **E** MDA-PCa-2b cells under 1 µM ENZ treatment, **F** LNCaP/AR cells under Veh (DMSO), **G** MDA-PCa-2b cells under Veh (DMSO), **H** PC3 cells under Veh (DMSO), and **I** DU145 cells under Veh (DMSO). All cells were transduced with Cas9 and annotated guide RNAs. All values were normalized to Day 0 and then to sgNT group; *n* = 3 independently treated cell cultures. *p* values were calculated using two-way ANOVA with a Bonferroni multiple-comparisons test. mean ± s.e.m. is presented. **J** Western blots of UBE2J1 and Cyclophilin B proteins, showing UBE2J1 KO and rescue in LNCaP/AR cells transduced with Cas9, annotated guide RNAs and wild type UBE2J1. **K** Bar plots represent the relative cell number fold change of LNCaP/AR cells transduced with Cas9, annotated guide RNAs and wild type UBE2J1; cells were treated with Veh (DMSO) or 10 µM ENZ, *n* = 3 independently treated cultures. All values were normalized to Veh group. *p* values were calculated using multiple t-test with Benjamini correction. mean ± s.e.m. is presented.

Despite the initial success of second-generation antiandrogens in treating PCa, unavoidable resistance emerges and significantly impairs patient outcomes [6]. Using an advanced ranking algorithm, MAGeCK, we re-analyzed our dataset from an in vivo library screen that identified genomic aberrations correlated with resistance [24]. Our analysis pointed to the depletion of ubiquitin-conjugating enzyme E2 J1 (UBE2J1) as a top candidate associated with antiandrogen (enzalutamide) resistance [24]. UBE2J1 also ranks as a top hit according to two additional factors: “the percentage of tumors in which shRNAs target a specific gene are enriched” and “the number of distinct hairpins enriched in resistant tumors”[24]. UBE2J1, a member of the E2 ubiquitin-conjugating enzyme family, is known for its crucial role in the endoplasmic reticulum (ER)-associated degradation pathway (ERAD) [25–27]. Although genomic depletion of UBE2J1 has been frequently observed in 5-15% of PCa patients (Fig. 1A), its role in PCa tumorigenesis remains largely unexplored. To elucidate the function of UBE2J1 in PCa, we utilized CRISPR/Cas9 to knockout (KO) UBE2J1 in two antiandrogen sensitive human PCa cell models: LNCaP/AR and MDA-PCa-2b (Fig. 1B). The clinical relevance of LNCaP/AR cells, which bear AR amplification, has been validated through the development of second-generation antiandrogens such as enzalutamide (ENZ) and apalutamide (APA) using this model [28]. It has been widely used to study resistance mechanisms to antiandrogens [24, 29, 30]. In fluorescence-activated cell sorting (FACS)-based competition assays using a cell mixture of wildtype (sgNT-RFP-positive) and UBE2J1-KO (sgUBE2J1-GFP-positive) cells (Fig. 1C), UBE2J1-KO conferred a significant growth advantage in the presence of enzalutamide in both LNCaP/AR and MDA-PCa-2b cell lines, as indicated by the increased percentage of sgUBE2J1-GFP cells compared to sgNT-RFP cells (Fig. 1D, E). However, in the absence of enzalutamide treatment, UBE2J1-KO LNCaP/AR and MDA-PCa-2b cells demonstrated only a minor growth advantage (Fig. 1F, G), which was not as marked as when the cells were subjected to antiandrogen therapy. These results may suggest that the growth benefit conferred by UBE2J1-KO only becomes necessary when the original AR signaling proves insufficient for the survival of PCa cells under the challenge of antiandrogens. This hypothesis is supported by the observation that no growth advantage was observed when UBE2J1 was KO in AR-independent cell lines, PC3 and DU145 (Fig. 1H, I). Furthermore, we validated the growth advantage granted by UBE2J1-KO using cell proliferation assays, which could be counteracted by restoring wild-type UBE2J1 expression (Fig. 1J, K). These findings suggest that UBE2J1-KO confers a profound growth benefit and resistance to antiandrogen treatment only when AR signaling is challenged and becomes insufficient in AR-dependent PCa cells.

**Fig. 2:**
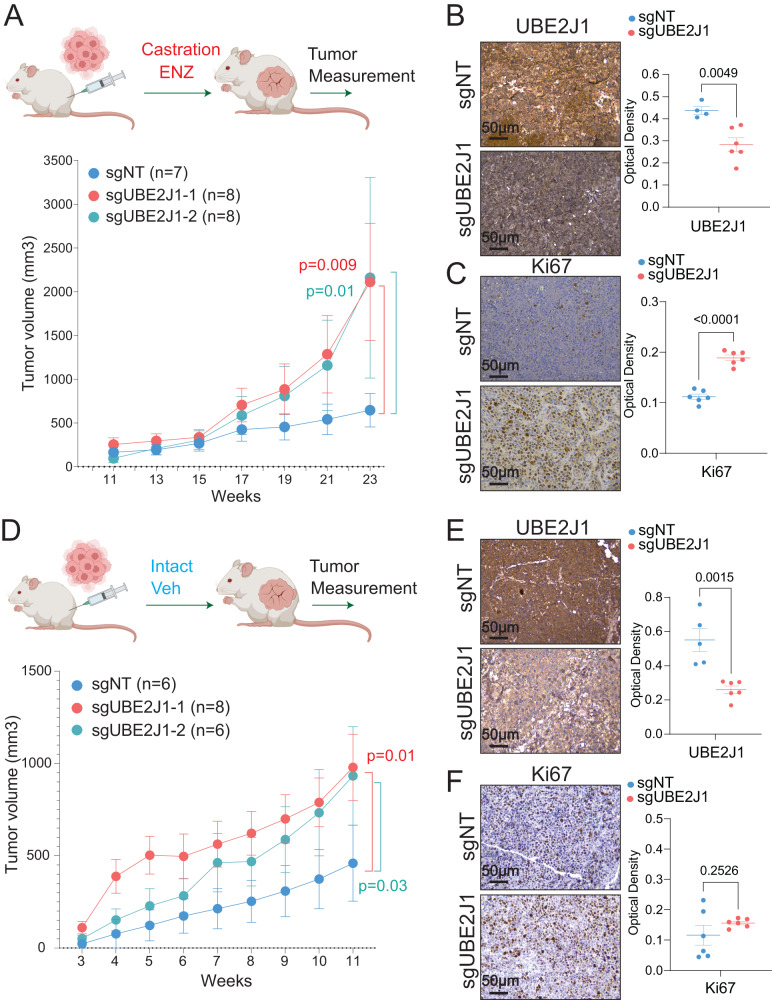
UBE2J1-loss promotes antiandrogen resistance in vivo*.* **A** Tumor growth curve of xenografted LNCaP/AR cells transduced with Cas9 and annotated guide RNAs in castrated mice. ENZ denotes enzalutamide treatment at 10 mg/kg from day 3 of grafting. The number (*n*) of tumors in each group was annotated. *p* values were calculated using two-way ANOVA with Bonferonni multiple-comparison test. Schematic figure was created with BioRender.com. **B**, **C** Immunohistochemical staining of **B** UBE2J1 and **C** Ki67 proteins on representative sgNT and sgUBE2J1 xenograft tumor slides from castrated mice in (**A**), scale bar represents 50 µm. Statistical analysis of representative pictures (*n* = 4–6) is presented, and *p* values were calculated using two-tailed *t*-test. **D** Tumor growth curve of xenografted LNCaP/AR cells transduced with Cas9 and annotated guide RNAs in intact mice. Veh denotes 0.5% CMC + 0.1% Tween 80 + 5% DMSO. The number (*n*) of tumors in each group was annotated. *p* values were calculated using two-way ANOVA with Bonferonni multiple-comparison test. Schematic figure was created with BioRender.com. **E**, **F** Immunohistochemical staining of **E** UBE2J1 and **F** Ki67 proteins on sgNT and sgUBE2J1 xenograft tumor slides from intact mice in (**D**), scale bar represent 50 µm. Statistical analysis of representative pictures (*n* = 4–6) is presented, and *p* values were calculated using two-tailed *t*-test. For all panels, mean ± s.e.m. is presented.

We further evaluated the role of UBE2J1 in PCa tumorigenesis and antiandrogen resistance in vivo using a LNCaP/AR xenograft model. Due to the amplified AR signaling in LNCaP/AR, the LNCaP/AR xenograft model stands as one of the best in vivo models resistant to androgen deprivation (ADT, castration) while retaining sensitivity to second-generation antiandrogens, like enzalutamide. Remarkably, xenografted UBE2J1-KO tumors exhibited increased growth under both castration and enzalutamide treatments (Fig. 2A), indicative of significant resistance to antiandrogens. Concurrently, we observed increased Ki67 staining in UBE2J1-KO tumors, suggesting that the loss of UBE2J1 may protect PCa tumors from the proliferation inhibition induced by antiandrogens (Fig. 2B, C). In line with our in vitro findings, xenografts of UBE2J1-KO cells into intact mice treated with a vehicle showed only moderately increased tumor growth (Fig. 2D), though not to the same extent as castrated mice treated with enzalutamide, as evidenced by similar levels of Ki67 staining (Fig. 2E, F). A possible explanation for this discrepancy between in vitro and in vivo findings is that the accumulation of AR and enhanced AR signaling may confer additional benefits for the growth of xenografted tumors in an in vivo setting. Taken together, these results support the hypothesis that the loss of UBE2J1 contributes to antiandrogen resistance in AR-dependent PCa.

### UBE2J1-loss confers therapy resistance through restoring AR signaling

**Fig. 3:**
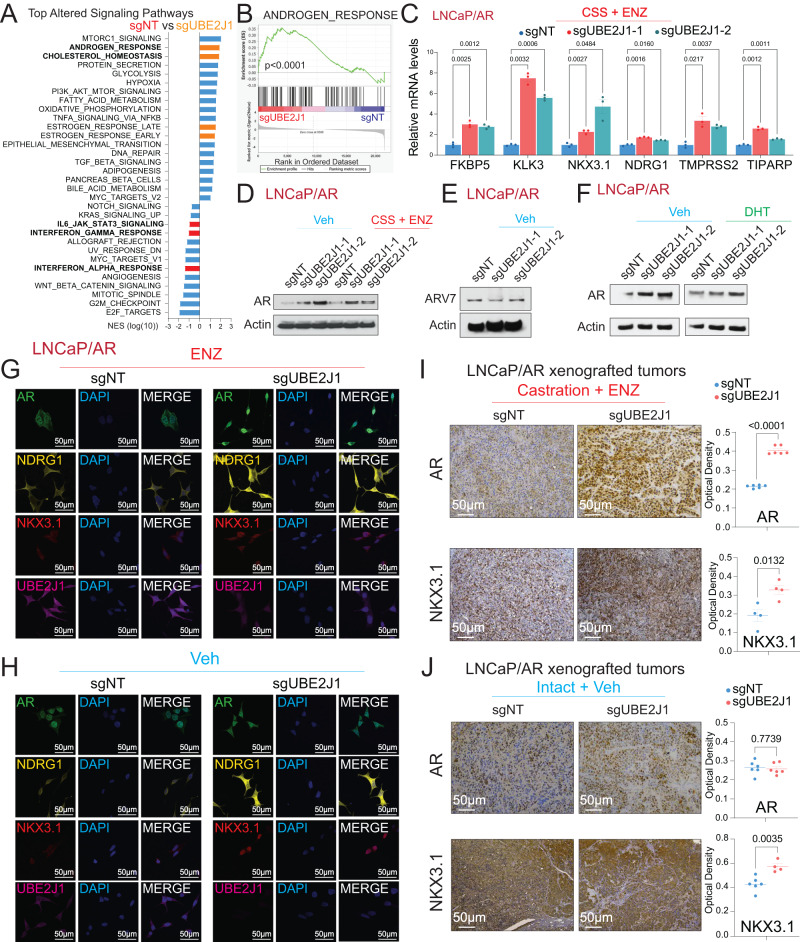
UBE2J1-loss leads to induction of AR and AR signaling. **A** GSEA Pathways analysis shows cancer-related signaling pathways significantly altered in UBE2J1-KO cells compared to wildtype cells, AR related and luminal lineage pathways were highlighted in orange and lineage specific pathways were highlighted in red. Reads from three biological replicates were used for analysis. **B** GSEA analysis represents the expression of hallmark androgen response gene signature in UBE2J1-KO cells compared to wild type. Reads from three biological replicates were used for analysis. **C** Relative gene expression of canonical AR target genes, measured by qPCR, in LNCaP/AR cells transduced with Cas9 and annotated guide RNAs, then treated with CSS + 10 µM ENZ for 5 days. *n* = 3 independently treated cultures, mean ± s.e.m. is presented. *p* values were calculated using two-way ANOVA with Bonferonni multiple-comparison test. **D** Western blots represent AR and Actin proteins in LNCaP/AR cells transduced with Cas9 and annotated guide RNAs, then treated with Veh (DMSO) in full serum or CSS + 10 µM ENZ for 5 days. **E** Western Blot represents AR-V7 and Actin proteins in LNCaP/AR cells transduced with Cas9 and annotated guide RNAs under Veh (DMSO). **F** Western Blot represents AR and Actin proteins in LNCaP/AR cells transduced with Cas9 and annotated guide RNAs, then treated with Veh (−DHT) or 10 nM dihydrotestosterone (DHT) for 48 h. **G**, **H** Representative immunofluorescence staining of LNCaP/AR cells transduced with Cas9 and annotated guide RNAs with annotated antibodies, scale bar represents 50 µm. Cells were treated with **G** 10 µM ENZ or **H** Veh (DMSO) for 7 days. **I** Immunohistochemical staining of AR and NKX3.1 proteins on sgNT and sgUBE2J1 xenograft tumor slides from castrated and ENZ treated mice, scale bar represents 50 µm. **J** Immunohistochemical staining of AR and NKX3.1 proteins on sgNT and sgUBE2J1 xenograft tumor slides from intact and Veh treated mice, scale bar represents 50 µm. For panels **G**, **H**, statistical analysis of representative pictures (*n* = 4–6) is presented, and *p* values were calculated using two-tailed t-test. For all panels, mean ± s.e.m. is presented.

To unravel the molecular mechanism facilitating antiandrogen resistance due to UBE2J1-loss, we probed the transcriptomic alterations upon UBE2J1-KO. To isolate the impact of UBE2J1-KO from that of AR inhibition by enzalutamide, we focused the RNA-Seq analysis on the vehicle-treated condition to identify the specific transcriptional alterations attributable to UBE2J1-KO, rather than those induced by drug-mediated AR suppression. Intriguingly, UBE2J1-KO resulted in an enriched AR signaling pathway, as well as other hormone-related signaling pathways, and conversely, diminished several signaling pathways associated with non-luminal and AR-independent lineages like JAK/STAT and interferon response signaling as shown by Gene Set Enrichment Analysis (GSEA) analysis (Fig. 3A, B) [29, 31, 32]. Consistent with the RNA-Seq findings, qPCR results confirmed the upregulation of canonical AR target genes upon UBE2J1-KO under androgen deprivation (charcoal-stripped serum, CSS-treated) and enzalutamide treatment (Fig. 3C). To examine the mechanism that led to this enhanced AR signaling, we first evaluated protein levels of AR and its common splicing variant, AR-V7, which is known to drive antiandrogen resistance [33]. Remarkably, AR protein levels were significantly elevated in UBE2J1-KO cells (Fig. 3D), suggesting an accumulation of AR proteins. However, using an AR-V7 specific antibody, AR-V7 protein levels remained unchanged in UBE2J1-KO cells (Fig. 3E), ruling out its role in conferring resistance in this context. These results also suggest that the ubiquitination sites on AR targeted by UBE2J1 are likely located in the C-terminal ligand-binding domain (LBD), which is absent in AR-V7. Furthermore, to evaluate the impact of UBE2J1-KO on AR under conditions of androgen induction, we treated LNCaP/AR cells with dihydrotestosterone (DHT) and observed a markedly more pronounced AR induction in the UBE2J1-KO cells (Fig. 3F). In accordance with these results, immunofluorescence (IF) staining revealed increased levels of AR proteins and heightened staining of the AR target genes NKX3.1 and NDRG1 in UBE2J1-KO cells treated with enzalutamide (Fig. 3G). In contrast, no significant changes were observed in vehicle-treated cells (Fig. 3H). Consistent with these in vitro findings, xenografted tumors from castrated mice treated with enzalutamide displayed elevated levels of AR and NKX3.1 proteins in UBE2J1-KO tumors compared to wildtype tumors (Fig. 3I). Contrastingly, no significant induction of AR and NKX3.1 proteins was observed in UBE2J1-KO tumors treated with vehicle (Fig. 3J), emphasizing the specific role of UBE2J1 in conferring antiandrogen resistance.

### UBE2J1-loss impairs AR degradation and leads to AR accumulation

**Fig. 4:**
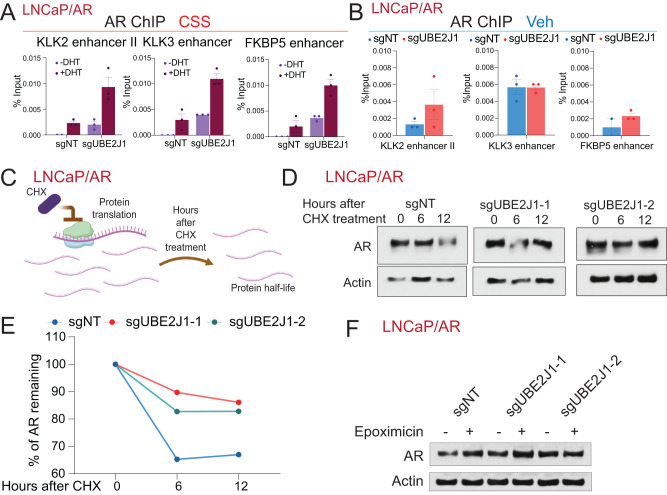
UBE2J1-loss leads to increased AR half-life and AR accumulation. **A** ChIP-qPCR of the genomic loci of canonical AR target genes in LNCaP/AR cells transduced with Cas9 and annotated guide RNAs, treated with Veh (−DHT) or DHT (+DHT) in charcoal stripped serum (CSS) media. **B** ChIP-qPCR of the genomic loci of canonical AR target genes in LNCaP/AR cells transduced with Cas9 and annotated guides RNAs, treated with Veh (DMSO). For (**A**), (**B**), *n* = 3 independently treated cell cultures and mean ± s.e.m. is presented. **C** Schematic figure represents the experimental setting of cycloheximide-based protein half-life assay. Schematic figure was created with BioRender.com. **D** Western blots represent AR and Actin proteins in LNCaP/AR cells transduced with Cas9 and annotated guide RNAs treated with cycloheximide (CHX) (30 µg/mL) for the annotated hours. **E** Dot plot represents quantification of western blot in (**D**) measuring AR half-life. **F** Western blot represents AR and Actin protein levels in LNCaP/AR cells transduced with Cas9 and annotated guide RNAs in the presence and absence of a proteasome inhibitor (10 µM Epoxomicin) for 6 h.

With the significant accumulation of AR proteins due to UBE2J1-KO, we hypothesized that this accumulation results in increased AR binding to its canonical target loci, consequently amplifying AR signaling and contributing to antiandrogen resistance. To test this hypothesis, we performed AR ChIP-qPCR analysis on both wildtype and UBE2J1-KO cells. Cells were cultured in CSS-medium to emulate an androgen-deprivation environment, then stimulated with AR ligand dihydrotestosterone (DHT). Notably, UBE2J1-KO led to a significant increase in AR binding at various canonical AR binding sites under CSS + DHT conditions (Fig. 4A), supporting our hypothesis. Furthermore, this induction of AR binding was less pronounced in vehicle-treated UBE2J1-KO cells (Fig. 4B), corroborating the specific role of UBE2J1 in restoring AR signaling. Collectively, our results demonstrate that the loss of UBE2J1 induces antiandrogen resistance by amplifying AR protein levels and restoring AR signaling in antiandrogen treated PCa cells.

Considering the accumulation of AR proteins and induction of AR target genes due to UBE2J1-loss, we aimed to uncover the molecular mechanism underlying this induction of AR and AR signaling. Given that UBE2J1 is an E2 ubiquitin-conjugating enzyme involved in protein ubiquitination and subsequent degradation [25–27], we postulated that the loss of UBE2J1 impairs the ubiquitination-dependent AR degradation, which results in the accumulation of AR proteins and restoration of AR signaling. To assess the protein degradation of AR, we treated the wildtype and UBE2J1-KO LNCaP/AR cells with cycloheximide (CHX), which halts protein synthesis and allows for the examination of protein half-life. After CHX treatment, we collected protein lysates at different time points and examined AR protein levels via western blot (Fig. 4C). Strikingly, AR protein half-life was significantly prolonged in UBE2J1-KO cells, demonstrating that UBE2J1-loss indeed slows down the degradation rate of AR (Fig. 4D, E). We then treated the LNCaP/AR cells with the proteasome inhibitor epoxomicin and assessed AR protein levels. Remarkably, the observed disparity in AR protein levels between UBE2J1-KO and wild-type cells was completely eradicated (Fig. 4F). This finding suggests that the AR accumulation induced by UBE2J1-KO is indeed dependent on proteasomal degradation.

**Fig. 5:**
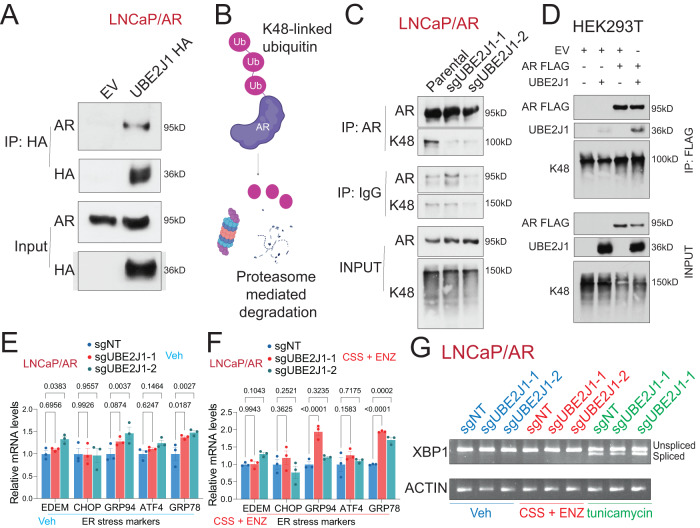
UBE2J1-loss leads to impaired AR ubiquitination and degradation. **A** Co-immunoprecipitation (Co-IP) of UBE2J1 (with HA-tag) and AR in LNCaP/AR cells transduced with empty vector (EV) or UBE2J1 HA-tag. **B** Schematic figure represents that K48-linked ubiquitination leads to degradation. Schematic figure was created with BioRender.com. **C** Co-immunoprecipitation (Co-IP) of AR and K48-ubiquitin in LNCaP/AR cells transduced with Cas9 and annotated guide RNAs. **D** Co-immunoprecipitation (Co-IP) of AR (with FLAG-tag) and UBE2J1 in HEK293T cells transduced with a combination of empty vector (EV), UBE2J1 and AR-FLAG. **E**, **F** Relative gene expression of canonical ER stress genes, measured by qPCR, in LNCaP/AR cells transduced with Cas9 and annotated guide RNAs and treated with **E** Veh (DMSO) in full serum media or **F** CSS + 10 µM ENZ for 5 days. *n* = 3 independently treated cultures. *p* values were calculated using two-way ANOVA with Bonferonni multiple-comparison test. mean ± s.e.m. is presented. **G** XBP1 splicing measured by qPCR and cDNA run on agarose gel. LNCaP/AR cells transduced with Cas9 and annotated guide RNAs were treated with Veh (DMSO) in full serum media, CSS + 10 µM ENZ for 5 days, or with tunicamycin (20 µg/mL) for 24 h.

As E2 ubiquitin-conjugating enzymes facilitate the attachment of ubiquitin to the target protein, we next probed whether UBE2J1 could interact with AR. Due to the absence of an IP-grade antibody for UBE2J1, we conducted co-immunoprecipitation (co-IP) experiments by overexpressing UBE2J1 with an HA-tag in parental LNCaP/AR cells. Notably, the co-IP experiment of AR and UBE2J1 demonstrated an interaction between the two (Fig. 5A), supporting the potential AR-E2 ubiquitin-conjugating function of UBE2J1. Considering that the most common pattern of ubiquitination associated with protein degradation is lysine 48 (K48)-linked ubiquitination [34–36], we conducted a co-IP of AR in wildtype LNCaP/AR and UBE2J1-KO cells to scrutinize the pattern of K48-linked ubiquitination in AR (Fig. 5B). Remarkably, UBE2J1-KO diminished K48-linked ubiquitination in AR (Fig. 5C). This finding is further validated by the increased K48-linked ubiquitination of AR-FLAG in the presence of UBE2J1 (Fig. 5D) in an overexpression system using HEK293T cells. Taken together, these data suggest that UBE2J1 modifies the ubiquitination pattern of AR, affecting AR half-life, and consequently leading to resistance.

Earlier studies have posited a role for UBE2J1 in ER-associated degradation pathway (ERAD), critical for clearing misfolded proteins in the ER [25–27]. To explore whether ER stress plays a part in the observed antiandrogen resistance induced by UBE2J1-KO, we carried out qPCR analysis to assess the expression of canonical ER stress markers [37]. However, we found no significant alterations in ER-stress related genes upon UBE2J1-KO (Fig. 5E, F), regardless of vehicle or antiandrogen treatment. Additionally, we treated wildtype and UBE2J1-KO cells with an ER stress inducer, tunicamycin (Fig. 5G), and assessed ER-stress induced XBP1 splicing [37]. Consistent with the gene expression data, we observed no significant alterations in XBP1 splicing regardless of vehicle or antiandrogen treatment (Fig. 5G). This further corroborates our hypothesis that the observed resistance to antiandrogen therapy is driven predominantly by the restoration of AR signaling rather than the known role of UBE2J1 in ERAD.

### Overcome antiandrogen resistance by restoring AR degradation

**Fig. 6:**
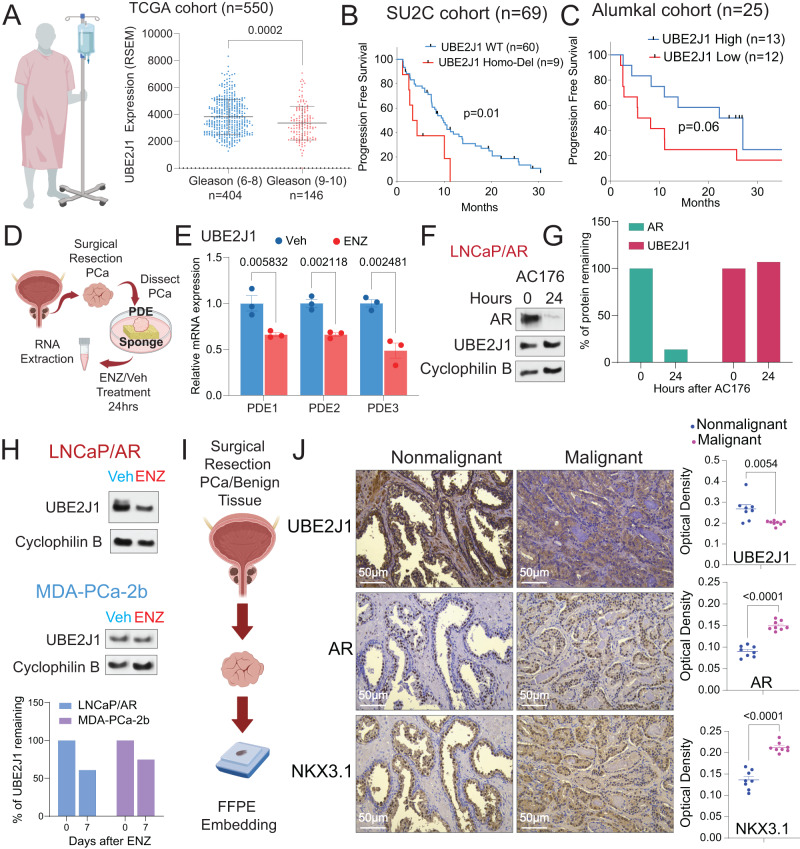
UBE2J1-loss correlates with poor clinical outcomes. **A** Expression of UBE2J1 in the high-grade tumors (Gleason score ≥ 9, *n* = 146 tumors) compared to the low-grade tumors (Gleason score ≤ 8, *n* = 404) in TCGA cohort. *p* values were calculated using two tailed *t*-test. Schematic figure was created with BioRender.com. **B**, **C** Kaplan–Meier curve represents the treatment duration on antiandrogen of patients with high or low expression of UBE2J1 in the **B** Stand up to Cancer (SU2C) cohort and **C** the Alumkal 2020 cohort. Abi abiraterone, ENZ enzalutamide, Apa apalutamide. *p* values were calculated with log-rank test. The number (*n*) of patients were annotated in each cohort. **D** Schematic figure represents the establishment of patient-derived explant (PDEs) models treated with Veh or 10 µM enzalutamide for 24 h. Schematic figure was created with BioRender.com. **E** Relative gene expression of UBE2J1 in independent patient-derived explants (*n* = 3) treated with Veh (DMSO) or ENZ for 24 h. *p* values were calculated using multiple t-tests with Benjamini correction. mean ± s.e.m. is presented. **F** Western blot represents AR, UBE2J1 and Cyclophilin proteins in LNCaP/AR parental cells treated with AR degrader (16 nM AC176) for the annotated hours. **G** Bar plot represents quantification of AR and UBE2J1 expression of western blot in panel (**E**). **H** Western blot represents UBE2J1and Cyclophilin B proteins in LNCaP/AR and MDA-PCa-2b parental cells treated with Veh (DMSO) or 10 µM ENZ for LNCaP/AR and 1 µM ENZ for MDA-PCa-2b for 7 days. Bar plot represents quantification of UBE2J1 expression of western blot. **I** Representative schematic of the establishment of de-identified formalin-fixed paraffin-embedded (FFPE) PCa samples. Schematic figure was created with BioRender.com. **J** Immunohistochemistry (IHC) staining of UBE2J1, AR and NKX3.1 proteins on PCa tumors and matched benign tissues. Scale bar represents 50 µm. Statistical analysis of representative pictures (*n* = 8) is presented, and *p* values were calculated using two-tailed *t*-test. For all panels, mean ± s.e.m. is presented.

Given the role of UBE2J1-loss in promoting antiandrogen resistance in preclinical models, we sought to evaluate the impact of UBE2J1-loss in various clinically relevant scenarios. We hypothesized that high-grade prostate cancer (PCa) patients, particularly those with Gleason scores of 9–10, who are more prone to develop resistance, would exhibit reduced expression of UBE2J1 compared to patients with low-grade tumors (Gleason scores 6–8) [38]. To validate this hypothesis, we analyzed patient data from the TCGA cohort segregated by tumor grade (Gleason score) and found a significant reduction in UBE2J1 expression in high-grade PCa patients compared to their low-grade counterparts (Fig. 6A), affirming the clinical relevance of UBE2J1-loss. To deepen our understanding of the role of UBE2J1-loss in antiandrogen resistance, we examined two metastatic Castration Resistant Prostate Cancer (mCRPC) patient cohorts: the Stand Up to Cancer (SU2C) and Alumkal 2020 cohorts [22, 23, 39]. These cohorts provided longitudinal clinical outcome data for patients undergoing treatment with second-generation antiandrogens. Consistent with our prior findings, we discovered that patients with UBE2J1-loss developed resistance to antiandrogen more rapidly than patients with wildtype UBE2J1 in the SU2C cohort (Fig. 6B). This observation was validated in the Alumkal 2020 cohort, where RNA-Seq analysis was performed before antiandrogen treatment. As we hypothesized, patients with lower UBE2J1 expression (below median) in their tumors developed resistance faster than those with higher UBE2J1 expression (above median) (Fig. 6C).

To further substantiate the clinical relevance of our discoveries, we utilized a series of patient-derived explants (PDEs), a well-validated clinical model for assessing ex vivo responses to antiandrogens (Fig. 6D) [24]. Notably, the expression of UBE2J1 in three independent PDEs consistently decreased upon treatment with enzalutamide (Fig. 6E). To further assess whether this reduction in UBE2J1 levels is a direct consequence of AR inhibition or an adaptive response, we evaluated the impact of acute AR inhibition on UBE2J1 levels. Utilizing an AR degrader, AC176, to diminish AR protein levels within a 24-hour period, we observed no significant alterations in UBE2J1 protein levels (Fig. 6F, G). These findings suggest that the observed reduction in UBE2J1 expression is likely attributable to the enrichment of pre-existing clones with relatively lower levels of UBE2J1, influenced by the stringent selection pressure exerted by enzalutamide in the PDEs. This hypothesis is further corroborated in both LNCaP/AR and MDA-PCa-2b cells, where a 7-day prolonged enzalutamide treatment resulted in reduced UBE2J1 protein levels (Fig. 6H). We then explored the relationship between UBE2J1-loss and AR signaling using de-identified formalin-fixed paraffin-embedded (FFPE) PCa samples (Fig. 6I). Immunohistochemistry (IHC) staining of these tumor samples showed a significant reduction of UBE2J1, accompanied by an increase in AR and AR target NKX3.1 proteins, in PCa compared to matched benign prostate tissue (Fig. 6J). Collectively, these results underscore the crucial role of UBE2J1-loss in conferring antiandrogen resistance, as validated through various clinically relevant models.

**Fig. 7:**
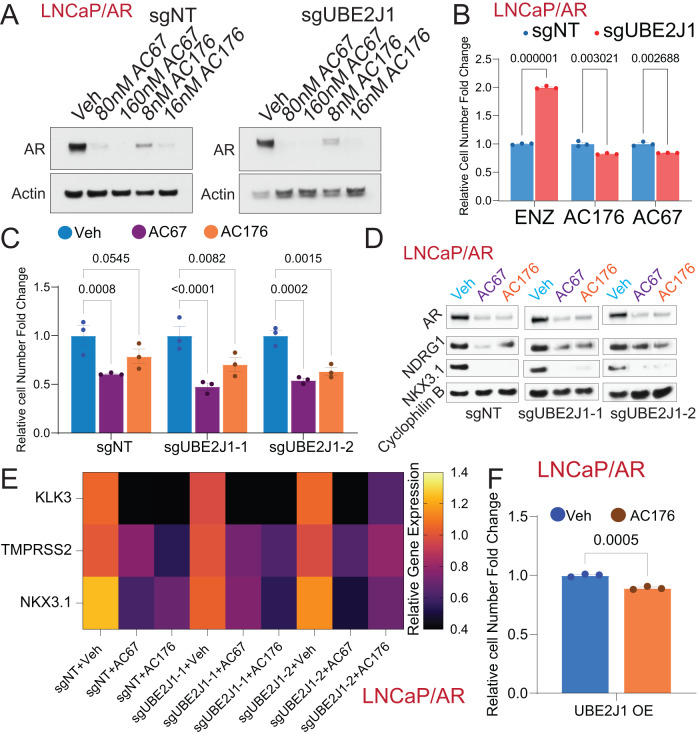
Restoring AR degradation suppresses antiandrogen resistance in UBE2J1-loss PCa. **A** Western blots represent AR and Actin proteins in LNCaP/AR cells transduced with Cas9 and annotated guide RNAs, treated with Veh (DMSO), CSS and AC67 or AC176 at the annotated concentrations. **B** Bar plot represents the relative cell number fold change for FACS-based competition assay of LNCaP/AR cells transduced with Cas9 and annotated guide RNAs, treated with 10 µM ENZ, CSS + 80 nM AC67 or CSS + 16 nM AC176. All values were normalized to sgNT group; *n* = 3 independently treated cell cultures. *p* values were calculated using multiple *t*-test with Benjamini correction. mean ± s.e.m. is presented. **C** Relative cell number fold change of LNCaP/AR cells transduced with Cas9 and annotated guide RNAs treated with Veh (DMSO), 80 nM AR degrader AC67 or 16 nM AC176 for 7 days; *n* = 3 independently treated cultures. All values were normalized to Veh group. *p* values were calculated using two-way ANOVA with Bonferonni multiple-comparison test. mean ± s.e.m. is presented. **D** Western Blot represents AR, NKX3.1, NDRG1 and Cyclophilin proteins in LNCaP/AR cells transduced with Cas9 and annotated guide RNAs then treated with Veh (DMSO), AR degrader 80 nM AC67 or AR degrader 16 nM AC176 for 4 days. **E** Heatmap represents AR target genes expression levels as measured by qPCR in LNCaP/AR cells transduced with Cas9 and annotated guide RNAs then treated with Veh (DMSO), AR degrader 80 nM AC67 or AR degrader 16 nM AC176 for 4 days. *n* = 3 independently treated cell cultures and mean is presented. **F** Relative cell number fold change of LNCaP/AR cells transduced with annotated overexpression (OE) treated with Veh (DMSO) or 16 nM AR degrader (AC176) for 7 days. *n* = 3 independently treated cultures, *p* values were calculated using two-tailed *t*-test and mean ± s.e.m. is presented.

**Fig. 8:**
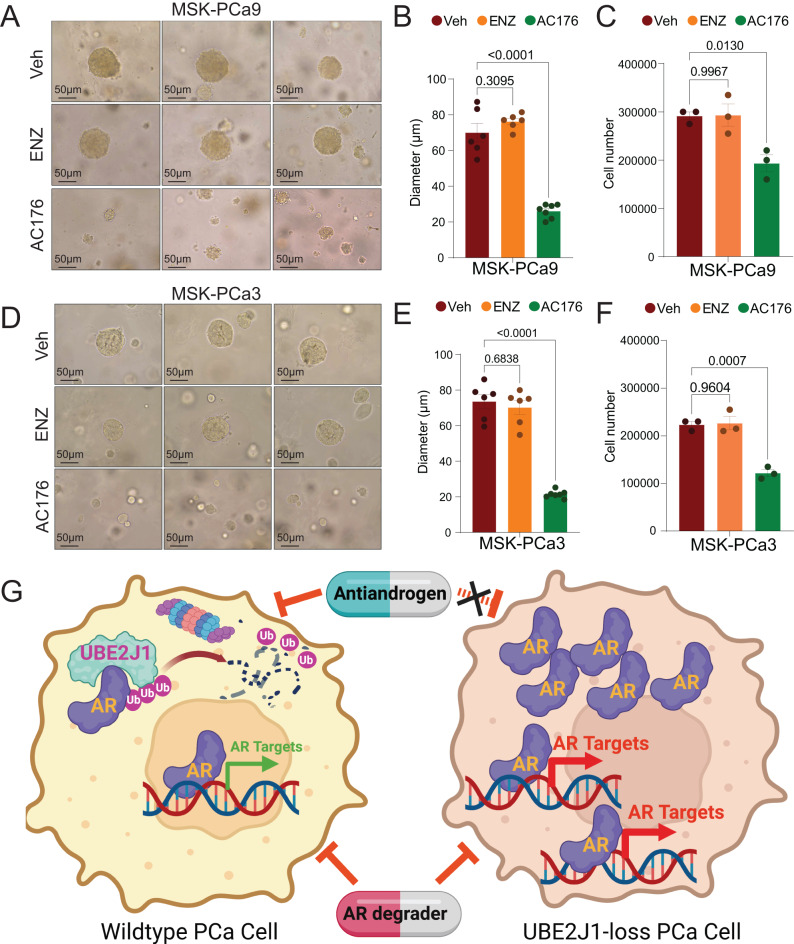
AR degraders suppresses antiandrogen resistance in PDOs. **A** Representative brightfield images of MSK-PCa9 PDO treated with Veh (DMSO), ENZ (1 µM) or AC176 (16 nM) for 10 days. Scale bar represents 50 µm. **B** Bar plot represents the diameter measurement of MSK-PCa9 PDO treated with Veh (DMSO) (*n* = 6), ENZ (*n* = 6) or AC176 (*n* = 7). **C** Bar plot represents the cell number count of MSK-PCa9 PDO treated with Veh (DMSO), ENZ or AC176. *n* = 3 independently treated PDO cultures. **D** Representative brightfield images for MSK-PCa3 PDO treated with Veh (DMSO), ENZ (1 µM) or AC176 (16 nM) for 10 days. Scale bar represents 50 µm. **E** Bar plots represent the diameter measurement of MSK-PCa3 PDO treated with Veh (*n* = 6), ENZ (*n* = 6) or AC176 (*n* = 7). **F** Bar plot represents the cell number count of MSK-PCa3 PDO treated with Veh (DMSO), ENZ or AC176. *n* = 3 independently treated PDO cultures. For all panels unless otherwise noted, mean ± s.e.m. is presented, and *p* values were calculated using one-way ANOVA with Bonferroni multiple comparison test. **G** Schematic figure represents the hypothetic function of UBE2J1 as the bona fide E2 ubiquitin-conjugating enzyme for AR ubiquitination in PCa and how its frequent loss in PCa leads to resistance to antiandrogens. Schematic figure was created with BioRender.com.

Given that the antiandrogen resistance in PCa caused by UBE2J1-loss stems from the impaired ubiquitination-dependent AR degradation, we hypothesized that restoring AR degradation could potentially reverse this resistance and inhibit the resistant growth of these PCa cells. To test this hypothesis, we employed two ubiquitination-based AR degraders, AC67 and AC176, known to induce strong AR degradation in LNCaP/AR cells [24]. We treated wildtype and UBE2J1-KO LNCaP/AR cells with these AR degraders to effectively restore AR degradation (Fig. 7A) and then evaluated the growth of these cells using FACS-based competition assays. Remarkably, both AR degraders completely reversed the growth advantage of UBE2J1-KO cells relative to wildtype cells (Fig. 7B), thus providing strong evidence for the efficacy of restoring AR degradation as a strategy to overcome resistance. Those results were further validated through cell proliferation assays (Fig. 7C). Furthermore, we analyzed both the expression and protein levels of canonical AR target genes in UBE2J1-KO and wildtype cells to assess AR activity. As anticipated, the levels of AR and its target genes were comparably suppressed by the AR degrader in both UBE2J1-KO and wildtype cells (Fig. 7D, E). This suggests that all observed phenotypic differences between UBE2J1-KO and wildtype cells are attributable to impaired AR degradation. Moreover, we overexpressed UBE2J1 in parental LNCaP/AR cells and observed that degraders could still inhibit the growth of UBE2J1-overexpressing (UBE2J1-OE) cells (Fig. 7F). To further evaluate this strategy in a more clinically relevant model, we employed two well-established, 3D-cultured patient-derived organoids (PDO) [40], which exhibit relatively low expression of UBE2J1 and are known for their high resistance to enzalutamide treatment due to enhanced AR signaling. Notably, despite their high resistance to enzalutamide, AR degrader AC176 significantly inhibited the growth of both MSK-PCa3 and MSK-PCa9 PDOs (Fig. 8A–F). These results support our hypothesis that UBE2J1-loss confers antiandrogen resistance by impairing AR degradation (Fig. 8G) and demonstrate the efficacy of restoring AR degradation in overcoming resistance, thereby laying the groundwork for future clinical studies.

## Discussion

Over the past two decades, targeted therapies have significantly improved survival rates for cancer patients, including those undergoing antiandrogen treatments for advanced PCa. However, resistance to antiandrogens often emerges, drastically limiting the clinical outcomes of these patients [7, 9, 24, 29, 30]. While AR-independent mechanisms have been suggested to induce antiandrogen resistance [24, 29, 30, 41], restoration of AR signaling remains the principal resistance mechanism for many patients [42]. Driver mutations in the AR ligand-binding domain have been linked to antiandrogen resistance, but only a small percentage of patients carry these mutations [6]. Additionally, AR variants, particularly AR-V7, have been suggested to be partially responsible for restoring AR signaling [5, 10, 33]. However, the exact mechanisms leading to the induction of AR proteins and downstream AR signaling in many patients remain largely elusive. In this study, we uncovered a previously uncharted mechanism that restores AR signaling by impairing the AR degradation machinery. We identified E2 ubiquitin-conjugating enzyme UBE2J1 as a key factor for AR ubiquitination in PCa. The frequent loss of UBE2J1 in 5-15% of PCa (cbioportal.org) [22, 23] disrupts AR ubiquitination and degradation, leading to an accumulation of AR proteins. This AR accumulation and induction allows UBE2J1-loss PCa cells to develop resistance to antiandrogen treatment. Interestingly, the frequency of homologous UBE2J1 loss is notably higher in primary cohorts like TCGA compared to more advanced metastatic cohorts such as SU2C and MCTP (Fig. 1A). Given that UBE2J1 functions as an E2 ubiquitin-conjugating enzyme responsible for AR degradation, loss of UBE2J1 would result in an accumulation of AR and enhanced AR-driven signaling. Consequently, primary PCa tumors, which are generally more reliant on AR signaling, would experience a greater growth advantage from UBE2J1 loss. These insights provide a deeper understanding of the molecular mechanisms underlying antiandrogen resistance.

Dysregulation of ubiquitination-based protein degradation is a frequent occurrence in many human cancers [14, 15, 17]. This dysregulation can originate from any of the three key players in the process: E1 ubiquitin activating enzymes, E2 ubiquitin-conjugating enzymes, and E3 ligases [16, 17, 20]. Recent studies have uncovered the roles of various E3 ligases in AR degradation. For instance, MDM2 has been shown to target AR for degradation in an AKT-dependent manner [43], while RNF6 has been shown to ubiquitinate AR, affecting its transcriptional activity [44]. Besides MDM2, CHIP has been suggested as one of the primary E3 ligases responsible for degrading AR protein [45]. Moreover, SPOP targets AR for degradation, and mutations in SPOP are associated with increased sensitivity to antiandrogens [46, 47]. Distinct from E3 ligases, E2 ubiquitin-conjugating enzymes determine the location of ubiquitin attachment on the target protein, thus dictating the fate of the protein and ubiquitin-substrate specificity in the ubiquitination process [18–21, 48]. However, the E2 ubiquitin-conjugating enzyme responsible for AR degradation has remained unclear. In this study, we are the first to identify UBE2J1 as an E2 ubiquitin-conjugating enzyme responsible for AR degradation. Our results show that the frequent loss of UBE2J1 in PCa leads to resistance to antiandrogen therapy through the restoration of AR signaling. Given the crucial role of E2 enzymes in tumorigenesis [20, 21], the identification of UBE2J1 as the key AR E2 enzyme represents one of the major novelties and significance of this study.

Despite the clinical success of antiandrogens, resistance to these agents inevitably emerges and severely limits the survival of patients with PCa. While numerous AR-independent mechanisms, including lineage plasticity [29, 30, 41], have been proposed as contributors to antiandrogen resistance, the restoration of AR signaling remains the most predominant mechanism in many patients [6]. Therefore, our results, which demonstrate the efficacy of restoring AR degradation using ubiquitination-based AR degraders, offer a potential effective strategy to counteract antiandrogen resistance. More importantly, as heterogeneous resistance mechanisms have been identified in different patients, many of which are AR-independent, it is vital to identify those patients who may develop antiandrogen resistance due to impaired AR degradation. In this context, our results, which suggest a correlation between UBE2J1-loss and antiandrogen resistance, propose that UBE2J1 could serve as an early biomarker for the identification of patients at risk of developing resistance due to impaired AR degradation and AR protein accumulation. These novel insights could lay the groundwork for future biomarker-based clinical trials and guide the development of effective therapeutic strategies to overcome antiandrogen resistance. This highlights the potential significance of our findings not just in enhancing our understanding of resistance mechanisms but also in improving the prognosis for patients affected by PCa.

## Methods

### Resource availability

#### Lead contact

Further information and requests for resources and reagents should be directed to and will be fulfilled by the Lead Contact, Dr. Ping Mu (Ping.Mu@UTSouthwestern.edu).

#### Material availability

All cell lines, plasmids, and other reagents generated in this study are available from the Lead Contact with a completed Materials Transfer Agreement if there is potential for commercial application.

#### Data and code availability

- All the described bulk RNA-seq data has been deposited in the Gene Expression Omnibus under the accession numbers GSE240305, reviewer token is otgrsqsszrydlib. Microscopy data reported in this paper will be shared by the lead contact upon request.
- All analysis in this manuscript was performed using open-source software. Bulk RNA-Seq analysis was done using the QBRC Bulk RNA-Seq pipeline and all code could be accessed: https://github.com/QBRC/QBRC_BulkRnaSeqDE. GSEA statistical analysis was carried out with the R package ‘fgsea’ (v1.14.0) in conjunction with the ‘Hallmark’ libraries from MsigDB.
- Any additional information required to reanalyze the data reported in this paper is available from the lead contact upon request.

### Experimental model and subject details

#### Cell lines

The parental LNCaP/AR cell line was obtained from the laboratory of C.L. Sawyers at Memorial Sloan Kettering Cancer Center (MSKCC). DU145 (HTB-81), PC3 (CRL-1435) and HEK293T (CRL-3216) cell lines were purchased from ATCC. LNCaP/AR and PC3 cells were cultured in RPMI 1640 medium supplemented with 10% Fetal Bovine Serum (FBS), 1% l-glutamine, 1% penicillin-streptomycin (p/s), 1% HEPES and 1% sodium pyruvate. DU145 and HEK293T cells were cultured in DMEM high-glucose medium supplemented with 10% FBS, 1% p/s and 1% l-glutamine. LNCaP/AR, DU145 and PC3 cells were passaged at a ratio of 1:6 every 3-5 days. HEK293T cells were passaged at a ratio of 1:8 every 3-5 days. The MDA-PCa-2b cell line was purchased from ATCC (CRL-2422) and cultured following the manufacturer’s instruction. More specifically, cells were cultured in Ham’s F 12 K (Kaighn’s) Medium supplemented with 20% FBS, 1% p/s, 25 ng/ml Cholera toxin (Sigma-Aldrich, C8052), 10 ng/ml mouse epidermal growth factor (Fisher Scientific, CB-40010), 0.0005mM O-phosphoethanolamine (Sigma-Aldrich, P0503), 0.1 ng/ml hydrocortisone (Sigma-Aldrich, H0135), 45 nM sodium selenite (Sigma-Aldrich, S9133) and 5 µg/mL human recombinant insulin (Thermo Fisher Scientific, 12-585-014). They were passaged at a ratio of 1:2 or 1:3 (depending on confluency) every 3–4 days and were cultured in Poly-d-Lysine (Gibco™, A3890401) coated plates. When LNCaP/AR cells were treated in charcoal stripped media (CSS), they were cultured in RPMI 1640 medium supplemented with 10% charcoal stripped serum (CSS), as well as all the other supplements mentioned above. All cell cultures were tested monthly for mycoplasma using the MycoAlert^TM^ Plus Mycoplasma Detection kit (Lonza, LT07-710). Cell lines were validated yearly using human short tandem repeat profiling cell authentication and compared to ATCC profiles.

#### CRISPR-Cas9 and overexpression plasmid

Lentiviral-based constructs were used for CRISPR-based KO and for overexpression of all genes in this study and were modified as described before [24, 29, 30]. Briefly, the All-In-One lentiCRISPRv2 (Addgene, 52961), pLKO5.sgRNA.EFS.RFP (Addgene, 57823) and pLKO5.EFS.GFP (Addgene, 57822) were used to generate the CRISPR and guide RNAs. A non-targeting RNA was used as empty control. The Benchling (https://benchling.com) guide RNA designing tool was used to design the guide RNAs. Sequences of guide RNAs are listed in Table S1. Key Resources. UBE2J1 (NM_016021.2), UBE2J1-HA (NM_016021.2) and AR-FLAG (NM_000044.6) were amplified from cDNA purchased from The McDermott Center for Human Growth and Development at UT Southwestern and cloned into a vector via restriction enzyme cloning. The UBE2J1 overexpression plasmid used for rescue assays was mutated at the PAM sequences as well as in the sequence were the guide RNA targets. All sequences were cloned into pLenti-CMV-P2A-blast vector (Addgene, 17486), or pLVX-IRES-PURO vector (Addgene, 107435).

#### Lentivirus preparation and CRISPR/overexpression cell line construction

The different cell lines utilized in this study were constructed by lentiviral infection as previously described [24, 29, 30], with minor modifications. HEK293T cells were seeded at a concentration of 1.5 × 10^6^ in 2 mL of medium in a 6-well plate 24 h before transfection. Plasmid and virus packaging vectors PspAX2 (Addgene, 12260) and VSV-G (Addgene, 138479), as well as Lipofectamine® 2000 (Thermo Fisher Scientific, 11668500) were diluted with OPTI-MEM I Reduced Serum Medium (Gibco™, 31985062) separately. After the diluted plasmid and the Lipofectamine® 2000 were mixed and incubated for 20 minutes at RT, the mixture was added to HEK293T cells in dropwise manner. The medium was changed 8–16 h after transfection. 24 and 48 h after the transfection, the virus containing medium was filtered with a 0.45 µm syringe filter and saved for transduction. LNCaP/AR, DU145 and PC3 cells were seeded at a concentration of 400,000 cells per well in 2 mL of medium in 6-well plates 24 h before the transduction. MDA-PCa-2b cells were seeded at a concentration of 800,000 cells per well in 2 mL of medium in a six-well plate coated with Poly-d-Lysine 48 h before the transduction. For the transduction the medium was replaced with medium containing 50% virus, 50% growth medium and 5 µg/mL polybrene (Millipore Sigma, TR-1003-G). The virus containing medium was replaced with regular growth medium after 24 h. Cells were selected with 2 µg/mL puromycin (InvivoGen, ANT-PR-1) for 4 days or 10 µg/mL blasticidin (Gibco™, A1113903) for 5 days.

#### In vivo xenograft experiment

All animal experiments were performed in compliance with the guidelines of the Animal Resource Center at UT Southwestern Medical Center. Animals were housed under humidity and temperature-controlled environment with a 12-h light/12-h night cycle in a pathogen free facility. The in vivo xenograft experiments were performed as previously described [24, 29, 30]. Briefly, 2 × 10^6^ LNCaP/AR cells were suspended in a solution where half the volume was Matrigel (BD Biosciences, 356237) and the other half was growth medium. We subcutaneously injected 100 µL into both flanks of 7-week-old castrated or intact SCID male mice. After 3 days of cell injection mice were gavage with 10 mg/kg Enzalutamide (daily) or vehicle (1% carboxymethyl cellulose, 0.1% Tween 80, 5% DMSO) (daily). All animals were separated into groups at random and tumor size was measured weekly with a digital caliper. The tumor cell injection and tumor treatment were performed by one researcher, while the tumor measurement and data analysis were performed by a different researcher to ensure that the studies were run in a blinded manner. The mice were monitored to minimize discomfort, distress, pain, or injury throughout the course of the xenograft experiments. Animals were removed from the study and euthanized if any signs of pain and distress were detected or if the tumor volume reached 2000 mm^3^. All procedures were performed in accordance with the recommendations of the Panel on Euthanasia of the American Veterinary Medical Association. The animal protocol was approved by the Institutional Animal Care and Use Committee (IACUC) of UT Southwestern Medical Center (protocol #2018-102461). Male CB17/lcr-*Prkdc*^scid^/lcrlcoCrl were purchased from Charles River. No statistical method was used to predetermine samples size, it was decided based on previously established protocols [24, 29, 30].

#### Cell growth and fluorescence-activated cell sorting (FACS)-based competition assays

For the cell growth assay in Fig. 1K, 9000 LNCaP/AR cells were plated in a 24-well plate in either vehicle (DMSO) or 10 µM ENZ in full serum medium. Cells were manually counted on day five using a hemocytometer. For the cell growth assay in Figs. 7C, 3000 LNCaP/AR cells were plated in a 24-well plate in either vehicle (DMSO), 80 nM AC67 or 16 nM AC176 in full serum medium. Cells were manually counted on day 7 using a hemocytometer. The fluorescence-activated cell sorting (FACS)-based competition assay was performed as described previously in [24, 29, 30]. Briefly, a 50/50 mixture was created with sgNT-RFP and sgUBE2J1-GFP cells and were either treated with vehicle (DMSO), 10 µM ENZ (LNCaP/AR), 16 nM AC176 (LNCaP/AR), 80 nM AC67 (LNCaP/AR) or 1 µM ENZ (MDA-PCa-2b) in full serum medium or treated with charcoal stripped serum (CSS) (LNCaP/AR). The percentages of RFP-positive and GFP-positive cells were measured using Attune NxT (version 4.2.1627.1) at Day 0 and the subsequent different days presented in the figures. Relative cell number fold change was calculated as previously described in [24, 29, 30]. To be certain that the results were not biased, the cell number and percentage were automatically measured by the Attune NxT Acoustic Focusing Cytometer. Bar plots visualizing the relative cell number fold change were created by Prism 10 Sotfware (https://www.graphpad.com/scientific-software/prism/). Three biological triplicates were used, mean ± s.e.m. is reported and experiments were repeated at least two times and achieved similar results. No data points were excluded.

#### Luminescent cell viability assay

CellTiterGlo® luminescent cell viability assay kit (Promega, 7570) was used to measure cell growth of LNCaP/AR cells transduced with UBE2J1 overexpression plasmid according to the manufacturer’s instruction. The cells were seeded in a 96-well plate and treated with Veh (DMSO) or AR degrader 16 nM AC176 for 7 days. 100 µL of CellTiterGlo® Reagent was added to each well and the contents were shaken for 12 min in an orbital shaker. The luminescence was then recorded with the Tecan Spark® Cyto plate reader. Treatments were conducted in triplicates and all experiments were repeated at least twice and achieved similar results. No data points were excluded and mean ± s.e.m. were reported.

#### Relative gene expression via RNA extraction and RT-qPCR

RNA was isolated from cells using TRIzol (Ambion, 15596018), and cDNA was made using SuperScript^TM^ IV VILO^TM^ Master Mix with ezDNase^TM^ (Thermo Fisher Scientific, 11766500) and 200 ng/mL RNA template, per manufacturer’s instructions. cDNA was amplified with 2× PowerUP^TM^ SYBR^TM^ Green Master Mix (Thermo Fisher Scientific, A25778). Each reaction was performed in triplicate, data was analyzed by the delta delta Ct method (2^−ΔΔCq^) and target genes’ expression was normalized to the expression of a house keeping gene. Bars visualizing the relative gene expression were created by Prism 10 Sotfware (https://www.graphpad.com/scientific-software/prism/) with expression fold change normalized to control cell lines. Three biological replicates were used and mean ± s.e.m. is presented. Experiments were repeated at least two times and achieved similar conclusions. No data points were excluded. XBP1 splicing assay was performed after cDNA amplification, the cDNA was run in a 2.5% agarose gel and visualized by ethidium bromide staining. Primers used for qPCR can be found in Table S1. Key Resources.

#### Protein detection via Western Blot

For western blots, protein was extracted from cell lysates with Radioimmunoprecipitation Assay lysis buffer (RIPA buffer, 150 mM sodium chloride, 1.0% NP-40 or Triton X-100, 0.5% sodium deoxycholate, 0.1% SDS, 50 mM Tris, pH 8.0) supplemented with protease and phosphatase inhibitors (Pierce^TM^, A32965) and incubated for 15 min on ice. The samples were then centrifuged at 20,000*g* for 10 min at 4 °C and the supernatant was collected. Protein quantification was performed using a Pierce BCA Protein Assay Kit (Pierce^TM^, 23225). Upon the addition of 5XSDS loading buffer containing 1% 2-mercaptoethanol (BME), protein lysates were boiled at 95 °C for 5 min. The samples were then resolved by SDS-PAGE using 1× NuPAGE MES SDS buffer (Invitrogen^TM^, NP0002) and then transferred for 2 h at 10 volts using 0.45 µm nitrocellulose membrane in 1× Blot Transfer buffer (Invitrogen^TM^, BT00061). After the transfer, membranes were blocked with 5% non-fat milk TBST for 1 h at RT before incubation with primary antibody overnight at 4 °C. After incubation with primary antibody, membranes were washed three times with 1× TBST and then incubated with secondary antibody for 1 h at RT. Membranes were washed with TBST three times and incubated with ECL (Thermo Scientific, 32209) or SuperSignal West Pico PLUS (Thermo Scientific, 34580) and developed to X-ray film in a dark room. The following antibodies were used for western blotting (also listed in Table S1. Key Resources): AR (D6F11) Rabbit mAb (Cell Signaling Technology, 5153), UBE2J1 (B-6) Mouse mAb (Santa Cruz Biotechnology, SC-377002), Cyclophilin B Rabbit mAb (Cell Signaling Technology, 43603), b-Actin (8H10D10) Mouse mAb (Cell Signaling Technology, 3700), Dykddddk Tag (D6W5B) Rabbit mAb (Cell Signaling Technology, 14793), HA-Tag (C29F4) Rabbit mAb (Cell Signaling Technology, 3724), K48 linkage Specific Polyubiquitin (D9D5) Rabbit mAb (Cell Signaling Technology, 8081), AR-V7 specific Rabbit mAb (Cell Signaling Technology, 68492), peroxidase AffiniPure goat anti-mouse IgG (H + L) (Jackson ImmunoResearch, 115-035-003) and peroxidase AffiniPure goat anti-rabbit IgG (H + L) (Jackson ImmunoResearch, 111-035-003). Followed manufacturer’s instructions for dilution of all primary antibodies. Dilutions of all secondary antibodies were 1:10,000.

#### Immunohistochemistry (IHC) and immunofluorescence (IF) staining

Tumors were collected from mice, washed with PBS and immediately fixed with 10% Neutral Buffered Formalin (StatLab Medical Products, 28600-1) at 4 °C overnight. Then the tumors embedded in paraffin by the UT Southwestern Tissue Management Shared Resource Core. After embedding, tumors were sectioned at 5 µm using a standard rotary microtome (Leica, Germany) and IHC staining was performed using standard protocol as previously described in [24, 29]. Briefly, slides were deparaffinized in xylene. Xylene was then removed with 100% ethanol and slides were hydrated in a series of ethanol dilutions until finally water was used. Sodium citrate buffer was utilized for antigen retrieval. Then endogenous peroxidase activity was blocked using 3% H_2_O_2_ in methanol. Slides were blocked with 3% BSA in PBST for 30 min at RT and incubated with primary antibody (AR (D6F11) Rabbit mAb (Cell Signaling Technology, 5153), Nkx3.1 (D6D2Z) XP® Rabbit mAb (Cell Signaling Technology, 92998), Ki 67 (D3B5) Rabbit mAb (Cell Signaling Technology, 9129), UBE2J1 Rabbit pAb (Sigma-Aldrich, HPA003509)), overnight at 4 °C. After incubating with primary antibodies, VECTASTAIN ABC HRP Kit (Peroxidase, Rabbit IgG) or Biotin-conjugated anti-rabbit IgG antibody (Jackson ImmunoResearch, 711-065-152) and peroxidase Streptavidin (Fisher Scientific, NC9705430) were used, followed by ImmPACT DAB Peroxidase (HRP) Substrate. Images were taken with the Leica DMi8 microscope and were quantified using Fiji ImageJ. Dot plots visualizing the quantification were created using Prism 10 Sotfware (https://www.graphpad.com/scientific-software/prism/). Immunofluorescence (IF) staining was performed as previously described in [24, 29]. Briefly, LNCaP/AR cells were plated on glass coverslips with Veh (DMSO) or 10 µM enzalutamide (pretreated for 5 days) and after 24 h they were fixed using 4% paraformaldehyde, permeabilized with 0.05% Triton X-100, blocked with 3% BSA in PBST and incubated in a humidified chamber with primary antibody (AR (D6F11) Rabbit mAb (Cell Signaling Technology, 5153), Nkx3.1 (D6D2Z) XP® Rabbit mAb (Cell Signaling Technology, 92998), Ndrg1 (D8G9) XP® Rabbit mAb (Cell Signaling Technology, 9485), UBE2J1 (B-6) Mouse mAb (Santa Cruz Biotechnology, SC-377002)) overnight at 4 °C. After washing with PBS, cells were incubated in the dark with Alexa Fluor 647 or Alexa Fluor 488 conjugated mouse or rabbit secondary antibody for 1 h at RT and then stained with DAPI. Images were acquired using a Zeiss LSM 700 confocal laser-scanning microscope. The following secondary antibodies were used for IF staining: Alexa Fluor 647-conjugated AffiniPure goat anti-mouse IgG (H + L) (Jackson ImmunoResearch, 115-605-003), Alexa Fluor 647-conjugated AffiniPure goat anti-rabbit IgG (H + L) (Jackson ImmunoResearch, 111-605-144), Alexa Fluor 488-conjugated AffiniPure goat anti-mouse IgG (H + L) (Jackson ImmunoResearch, 115-545-003) and Alexa Fluor 488-conjugated AffiniPure goat anti-rabbit IgG (H + L) (Jackson ImmunoResearch, 111-545-003). Used the following dilutions for primary antibodies for IHC: AR 1:400, UBE2J1 1:400, Ki67 1:400 and NKX3.1 1:200. Used the following dilutions for primary antibodies for IF: AR 1:600, NKX3.1 1:250, NRDG1 1:200 and UBE2J1 1:50. Dilutions of all secondary antibodies for IF were 1:500. The pictures were coded to blind researchers to treatment or genotype groups prior to data analysis to avoid bias.

#### AR ChIP-qPCR

ChIP-qPCR experiments were performed as previously described in [30]. Control and UBE2J1 KO LNCaP/AR cells were grown in RPMI-1640 media supplemented with 10% Charcoal Stripped FBS (CSS) or regular FBS for 3 days. Cells were plated in a 15 cm dish a day prior and cells grown in CSS were treated with 10 nM Dihydrotestosterone (DHT) or Veh for 2 h. Cells were then fixed with 1% paraformaldehyde for 15 min and quenched with 0.125 M glycine for 5 min. Then the cells were rinsed with cold 1× PBS twice and lysed in 300 µL ChIP lysis buffer with protease and phosphatase inhibitors (Pierce^TM^, A32965). Chromatin was sonicated by Bioruptor® Pico to obtain an average length of 200–300 base pairs. 1% of the sample was saved as input, and the rest was incubated with 5 µg Anti-AR Antibody (Abcam, ab108341) or 5 µg Normal Rabbit IgG Polyclonal Antibody (Millipore Sigma, 12-370) overnight at 4 °C. After overnight incubation, the samples were incubated for 4 h at 4 °C with Dynabeads Protein G (Fisher Scientific, 10-003-D). The beads were washed with standard wash buffers (low-salt, high-salt and LiCl wash buffer) and finally with TE buffer. The chromatin was eluted from the beads, followed by decrosslinking using 0.2 M NaCl at 65 °C for 4 h. Input and ChIPed DNA were purified with a MiniElute PCR purification kit (Qiagen, 28004) and the concentration was determined by Qubit. For ChIP-qPCR, DNA was amplified with 2× PowerUP^TM^ SYBR^TM^ Green Master Mix (Thermo Fisher Scientific, A25778). Triplicates were conducted for each reaction and the enrichment percentage was calculated using the input. Three biological replicates were used and mean ± s.e.m. is presented. Experiments were repeated at least 2 times and similar conclusions were achieved. No data points were excluded. Primer sequences are listed in Table S1. Key Resources.

#### Bulk RNA-Seq preparation and analysis

RNA was extracted from LNCaP/AR cells using TRIzol (Ambion, 15596018). The extracted total RNAs were sent to BGI Genomics Global to perform bulk RNA-Seq. RNA-Seq libraries were then prepared using the stranded Illumina TruSeq mRNA kit. The preparation began with 500 ng of total RNA and included 10 cycles of PCR amplification. These barcoded RNA-Seq libraries were run as paired-end, 50-nucleotide reads on an Illumina HiSeq 2500 and were selectively filtered by poly(A). The alignment of the reads to the human reference genome (GRCh38) was executed using STAR (v2.7.2b)72 [49], while FeatureCounts (v1.6.4)73 [50] was employed for gene counts, biotype counts, and rRNA estimation. The differential expression analysis was conducted using the R package DEseq2 (v1.26)74 [51], with selected cutoff values of an absolute fold change greater than 2 and a false discovery rate of less than 0.1. The differentially expressed genes were then identified, and GSEA was performed using the R package fgsea (v1.14.0) in conjunction with the ‘Hallmark’ libraries from MsigDB [52].

#### Patient derived explants (PDE), patient derived organoids (PDO) and FFPE samples

PDE models were established from prostate cancers (male) in the Raj laboratory, around 1 mm^3^ samples were embedded in a sponge and cultured in RPMI-1640 media with 10% FBS, 1% penicillin-streptomycin, 0.01 mg/mL hydrocortisone and 0.01 mg/mL insulin [29]. PDEs were treated with 10 µM enzalutamide or DMSO for 24 h and then RNA was extracted. RT-qPCR was performed and analyzed using the protocol mentioned in *Relative gene expression via RNA extraction and RT-qPCR*. PDO models were established in the Chen laboratory [53]. PDOs were cultured in 3D Matrigel with human organoid medium and were split at a 1:3 ratio every 7 days using trypsin or a sterile glass pipette. When treated with Veh (DMSO), 1 µM ENZ or 16 nM AC176, these organoids were cultured in typical human organoid medium supplemented with drugs. 1 µM ENZ was used as the concentration because patients-derived organoids in 3D culture are more sensitive to enzalutamide. Images were acquired with Leica DMi8 microscope. Diameter was quantified using Fiji ImageJ. Bar plots visualizing the measurements were created using Prism 10 Sotfware (https://www.graphpad.com/scientific-software/prism/). Benign prostate tissue and PCa frozen samples were established in Strand and Raj laboratories. De-identified human PCa Formalin-Fixed Paraffin Embedded (FFPE) slides were purchased from UT Southwestern Tissue Management Shared Resource Core and IHC was performed as mentioned above in *Immunohistochemistry (IHC) and Immunofluorescence (IF) staining*. The pictures were coded to blind researchers from treatment or genotype groups prior to data analysis to avoid bias.

#### Co-immunoprecipitation (Co-IP)

Co-immunoprecipitation was performed as previously described in [24] with some modifications. For western blotting in Fig. 5D 1.0 × 10^6^ HEK293T cells were seeded into each well of a six-well plate, 2 µg plasmids (pLVX-IRES-PURO-FLAG-AR, pLenti-CMV-P2A-blast UBE2J1, pLenti-CMV-P2A-blast EV) were mixed with 12 µL Lipofectamine® 2000 in 200 µL OPTI-MEM I Reduced Serum Medium for 20 min at RT. The mixture was added in a dropwise manner to the cells and swirled to mix. After 24 h the cells were lysed in 200 µL ice-cold mammalian IP lysis buffer (25 mM Tris-HCl, pH 7.4, 150 mM NaCl, 1 mM EDTA, 10% NP40, 10 mM N-ethylmaleimide) supplemented with 1× Pierce protease and phosphatase inhibitors (Pierce^TM^, A32965) for 30 min with constant rotation at 4 °C. The samples were then centrifuged at 20,000*g* for 10 min at 4 °C, 40 µL of the supernatant were collected for input (whole cell lysate). The rest of the supernatant was incubated with 5 µL of pre-washed and pre-blocked Anti-FLAG® M2 Magnetic Beads (Sigma-Aldrich, M8823) (blocked with 3% BSA in PBS for 30 min at 4 °C with constant rotation) overnight at 4 °C with constant rotation. The next day the magnetic beads in combination with any of the targets were isolated by magnet (Invitrogen^TM^, 12321D). Beads were washed with cold IP lysis buffer supplemented with 1 mM PMSF AND 0.5 mM DTT 3 times. After the final wash, IP and input samples were boiled with 1×SDS loading buffer + 1% BME at 95 °C for 5 min. For Fig. 5A LNCaP/AR cells transduced with pLenti-CMV-P2A-blast or pLenti-CMV-P2A-blast UBE2J1 HA were seeded in a 10 cm plate and then followed the same steps as previously mentioned with some changes. Cells were lysed with 1 mL of ice-cold lysis buffer (same as previously mentioned). The supernatant was incubated with 15 µL pre-washed Pierce^TM^ Anti-HA Magnetic Beads (Thermo Fisher Scientific, 88836) pre-blocked with 3% BSA in PBS for 30 min at 4 °C with constant rotation, then followed the same steps previously mentioned. For Fig. 5C LNCaP/AR cells transduced with Cas9 and annotated guides were seeded in a 10 cm plate and lysed with 1 mL ice-cold lysis buffer (same as before). Supernatant was incubated with 0.4 µL rabbit IgG (Millipore Sigma, 12-370) or 8 µL AR (D6F11) Rabbit mAb (Cell Signaling Technology, 5153). After overnight incubation at 4 °C with constant rotation, samples were incubated with 15 µL Pierce Protein A/G Magnetic Beads (Pierce^TM^, 88802) for 3 h at 4 °C with constant rotation. After that, followed the same steps previously mentioned. All experiments were repeated at least twice and achieved similar conclusions.

#### Statistical methods

Statistical details of each experiment were shown in figure legends. Two-tailed *t*-test with Welch’s correction for unequal variances was used to compare two groups of independent datasets that fit normality and homoscedasticity. When normality and homoscedasticity were not satisfied, Mann Whitney *U* Test (nonparametric Wilcoxon Rank Sum Test) was used when comparing gene expressions between two patients’ groups. For comparisons involving more than two groups, one-way or two-way ANOVA and Kruskal-Wallis nonparametric ANOVA were used as appropriate. mean ± s.e.m were reported, and *p* values were calculated and adjusted for multiple comparisons (Bonferroni or Benjamini correction) when applicable. For survival studies, the Kaplan-Meier method was used to estimate and plot the survival curve, and the log-rank test analysis was used to evaluate differences in survival data among different groups. For all in vitro experiments, three biological replicates were performed except when noted differently in figure legends.

#### Analysis of human prostate cancer dataset

Processed 444 SU2C metastatic prostate cancer patient cohort data, including RNA-Seq data and enzalutamide/abiraterone treatment data were downloaded from cBioPortal (RRID: SCR_014555, http://www.cbioportal.org/) [22, 23]. The cohort of 69 patients with clinical response data were examined for progression free survival. The probability of treatment duration figure was generated by prism 10 using Mantel-Cox test. The TCGA PRAD dataset, including gene expression FPKM values of 498 primary tumors and 52 normal tissue controls, was downloaded from the Genomic Data Commons Data Portal (https://portal.gdc.cancer.gov/). The Akumal 2020 cohort was obtained from the clinical trial Genetic and Molecular Mechanisms in Assessing Response in Patients with Prostate Cancer Receiving Enzalutamide Treatment [39]. The cohort included 25 patients with mCRPC who had not previously received enzalutamide that had data from RNA-Sequencing (RNA-Seq). The probability of treatment duration figure was generated by prism 10 using Mantel-Cox test.

### Supplementary information

Supplemental Table
